# Proceedings of the Terrell Medical Society

**Published:** 1887-10

**Authors:** 


### SOCIETY NOTES.

### PROCEEDINGS OF THE TERRELL MEDICAL SOCIETY.

STATED MEETINGS 1st AND 3rd MONDAY'S IN EACH MONTH.

Dr. Geo. A. Nelson in the chair.

Dr. John Inabnit read a paper on diseases of the uterus, setting forth a preference for caustic applications to the os tincæ, as well as the cervix—and also favored the application of this agent to the os of a gravid uterus, in cases of persistent vomiting.

Dr. Nelson entered his protest to this procedure in the gravid uterus, and stated as his conviction, that uterine catarrh is frequently induced by the too frequent use of the vaginal douche, as the fluid so injected often passes directly into the uterine cavity —and that the center hole in such syringes should be closed;— he was confident that by its use and the use of such agents as argenti nitras, caustics, etc., thus used, abortion was frequently induced.

Dr. Strain was of the opinion that there were agents, equally as effective and void, to a greater extent, of danger, than those already mentioned, and concluded that generally these agents were more frequently used because of their being near at hand; but that he preferred a more conservative course of treatment.

Dr. Webb read a paper upon abortion, and asked whether or not it was criminal to induce an abortion, otherwise than to save the life of the mother ?

Dr. Inabnit contended that it was.—

Dr. Nelson held that circumstances should be brought to bear upon the case; if an intelligent and chaste young woman should be deceived by her lover, whithout any interference she would be for ever disgraced and lead the life of the abandoned, whereas to interfere, she could, or might, be restored to her family and society, becoming both an ornament and a useful member of the same. Of course, he did not propose to establish this as a rule in general, for he knew full well that there are enough designing women who would abuse this exception—as applied to special cases.

Dr. Webb seemed inclined to think it was more or less a hazardus experiment, and should be styled criminal, and so held.

Dr. Anthony thought that circumstances should be considered, and that in such cases as really demanded protection from a life of shame and misery, the physician would be not only justified, but lauded in his virtuous endeavors, to snatch as it were, a brand from the burning.

Dr.Smiley was of the opinion that to induce an abortion to protect a woman's virtue or character, which had been betrayed, if a crime, was less crime than to send the woman headlong to ruin.

Dr. Monday read a paper upon the subject viz: "Shall a physician pander to the whims of his patients?"

Dr. Smiley said that he hoped that no intelligent physician would be found, who would sacrifice the recognized course of duty to his patient, simply to satisfy a whim which is born of a disordered mind, but should be respectfully passed by.

Dr. Smiley read a paper on Puerperal Eclampsia, in which he favored copious blood letting as the sheet anchor treatment.

Dr. Nelson read a paper from several cases of cholera infantum and demonstrated what satisfactory results followed the ice water enema after each motion from the bowels—it lessened the inflammatory movement, induced quiet, altered the character of the passages and lessened their frequency.—The Doctor stated that at the time to which he referred, the atmosphere was seriously contaminated, but notwithstanding he lost not a single case, having used but little internal treatment.

Dr. Monday read a paper upon the oxytoccic effect of quinine, claiming that this drug would induce abortion, when given in full doses at regular intervals.

Dr. Smiley held to the opposite view as he had dispensed the drug in several instances in full doses of gr. xx every 3rd hour with a view to induce abortion and prove the drug to be capable of inducing abortion, but met with negative results.

Dr. Wallace said that he was not willing to risk its effects and that he always guarded such tendency if any there should be in the drug—usually with pulv-doveri—but that he prefered digitalis in such cases as were exposed to any danger.

